# Supplementary material for: RNA is a key component of extracellular DNA networks in Pseudomonas aeruginosa biofilms
Source: Nat Commun. 2023 Nov 27;14:7772. doi: 10.1038/s41467-023-43533-3 (PMC10682433; doi:10.1038/s41467-023-43533-3)
Supplement: Supplementary file 1 — Supplementary Information [file 41467_2023_43533_MOESM1_ESM.pdf]

Supplementary Information

RNA is a key component of extracellular DNA networks in *Pseudomonas* biofilms

Sudarsan Mugunthan[1], Lan Li Wong[1], Fernaldo Richtia Winnerdy[2], Stephen Summers[1], Muhammad Hafiz Bin Ismail[3], Yong Hwee Foo[1,4], Tavleen Kaur Jaggi[5], Oliver W. Meldrum[5], Pei Yee Tiew[6], Sanjay H. Chotirmall[5,7], Scott A. Rice[1,8**], Anh Tuân Phan[9], Staffan Kjelleberg[1,10,11*], Thomas Seviour[1,12*].

[1] Singapore Centre for Environmental Life Sciences Engineering, Nanyang Technological University, Singapore, 637551.

[2] School of Medicine, University of California, San Francisco, CA 94158, USA

[3] Environmental Health Institute, National Environmental Agency, Singapore 138667

[4] Institute for Digital Molecular Analytics and Science (IDMxS), Nanyang Technological University, Singapore, 636921

[5] Lee Kong Chian School of Medicine, Nanyang Technological University, Singapore, 636921.

[6] Department of Respiratory and Critical Care Medicine, Singapore General Hospital, Singapore

[7] Department of Respiratory and Critical Care Medicine, Tan Tock Seng Hospital, Singapore

[8] The iThree Institute, University of Technology Sydney, Sydney, 2007, Australia.

[9] School of Physical & Mathematical Sciences, Nanyang Technological University, Singapore, 637371,

[10] School of Biological Sciences, Nanyang Technological University, Singapore, 637551, Singapore.

[11] School of Biological, Earth and Environmental Sciences, University of New South Wales, 2052, Australia.

[12] Centre for Water Technology (WATEC), Department of Biological and Chemical Engineering, Aarhus University, Ole Worms Allé, 8000, Aarhus C, Denmark.

** Current position: CSIRO, Agriculture and Food, Westmead and Microbiomes for One Systems Health

*Corresponding authors Thomas Seviour, Staffan Kjelleberg.

Email: twseviour@bce.au.dk; laskjelleberg@ntu.edu.sg

Supplementary Figure 1:

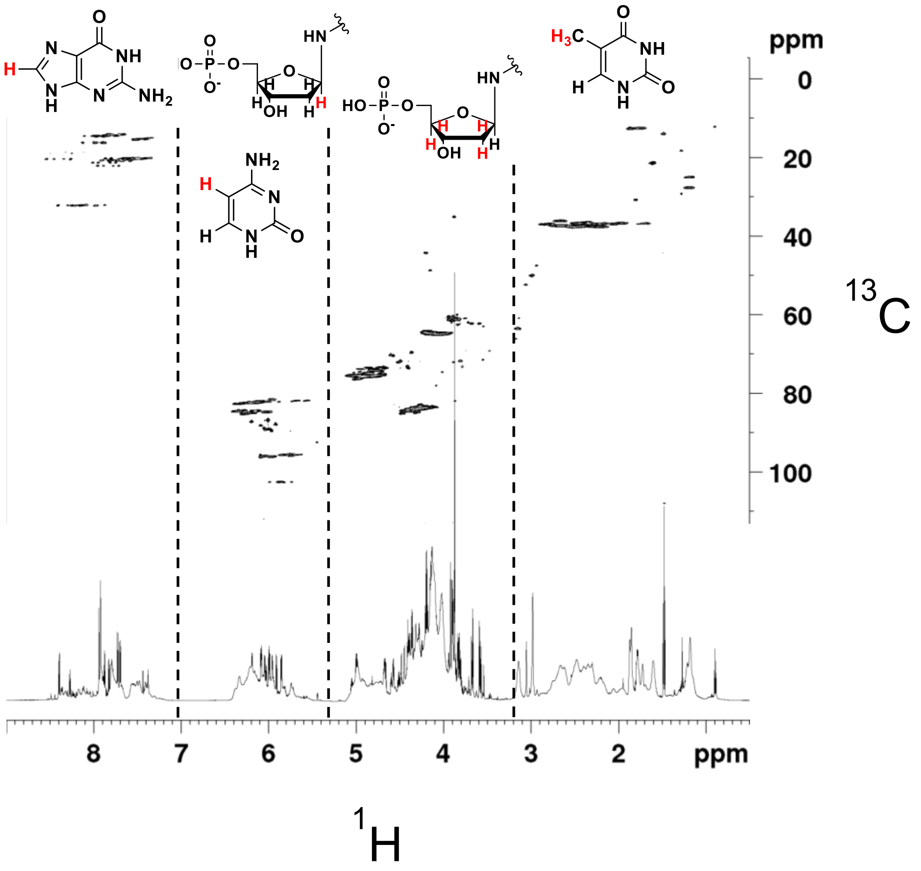

Supplementary figure 1: ^{1}H-^{13}C HSQC-TOCSY and 1-D ^{1}H NMR spectra of *Pseudomonas aeruginosa* PAO1 extracellular NA gel isolate following alkalinization (0.1 M NaOD, 10 mg/mL, 55°C, 2 h) at 25°C showing a distribution of ^{1}H-^{13}C HSQC-TOCSY cross peaks that is consistent with the presence of nucleic acids and the absence of proteins and hexose-based sugars.

Supplementary Figure 2:

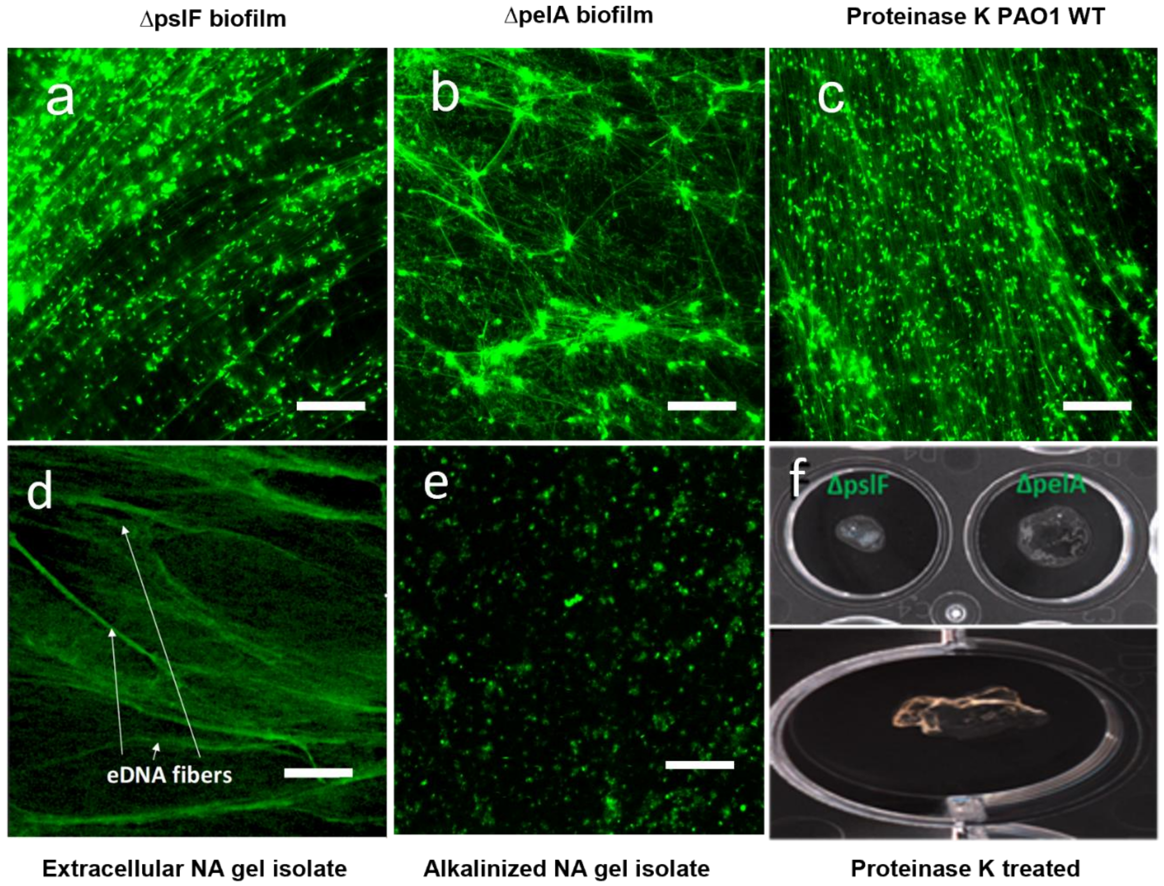

Supplementary Figure 2: Confocal micrograph of five-day old *P. aeruginosa* (n=3) (a) ΔpslF polysaccharide mutant, (b) ΔpelA polysaccharide mutant and (c) proteinase K treated wildype, stained with eDNA specific stain TOTO-1 showing eDNA fibres in green. Confocal micrograph of purified (d) untreated extracellular nucleic acid (NA) gel isolate and (e) alkalinized extracted from five-day old wildtype *P. aeruginosa* stained with eDNA specific TOTO-1 showing eDNA fibres in green. The scale bars represent 10 µm. (f) Gel forming purified extracellular nucleic acid extracted from five-day old ΔpslF, ΔpelA *P* and proteinase K treated *P. aeruginosa* biofilms respectively. All experiments were performed in biological triplicates.

Supplementary Figure 3:

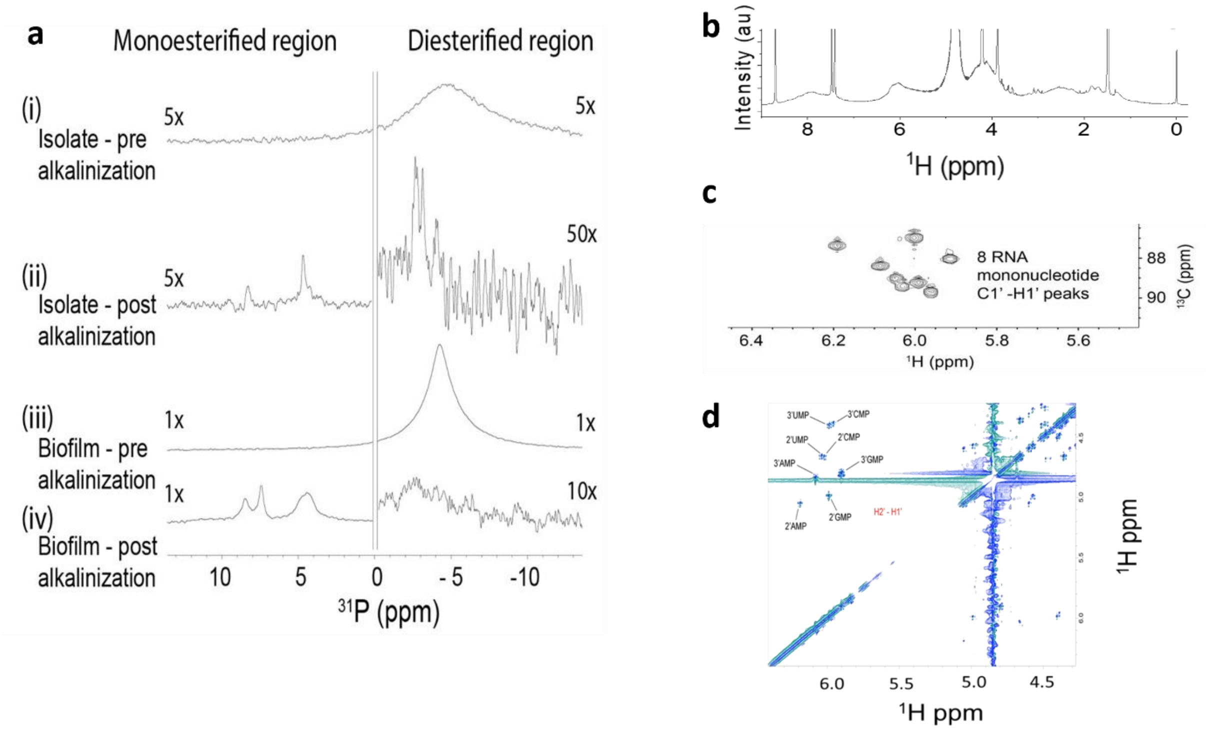

Supplementary Figure 3: (a) ^{31}P NMR spectra at T = 25°C of extracellular eDNA gel isolate (i), alkalinized and lyophilized eDNA gel isolate (ii), *Pseudomonas aeruginosa* biofilm (iii) and alkalinized and lyophilized *P. aeruginosa* biofilm (iv) showing the presence of diesterified phosphate peaks and the absence of monoesterified phosphate peaks for both eDNA gel isolate and biofilm in double distilled water, and the coexistence of diesterified and monoesterified phosphate peaks for both samples after alkalinization. This indicates that alkalinization of the matrix results in RNA transesterification. (b) ^{1}H NMR spectra of *P. aeruginosa* biofilm extracellular nucleic acid (NA) gel isolate in D_2O at 25°C. (c) ^{1}H-^{13}C HSQC spectrum of extracellular nucleic acid (NA) gel isolate (0.1 M NaOD, 10 mg ml^{-1}) from *P. aeruginosa* biofilm at 25°C identifying the eight monoribonucleic acid ribose spin systems. (d) ^{1}H-^{1}H COSY NMR spectrum of *P. aeruginosa* biofilm extracellular nucleic acid (NA) gel isolate following alkalinisation (0.1 M NaOD, 10 mg ml^{-1}, 55°C, 2 h).

Supplementary Figure 4:

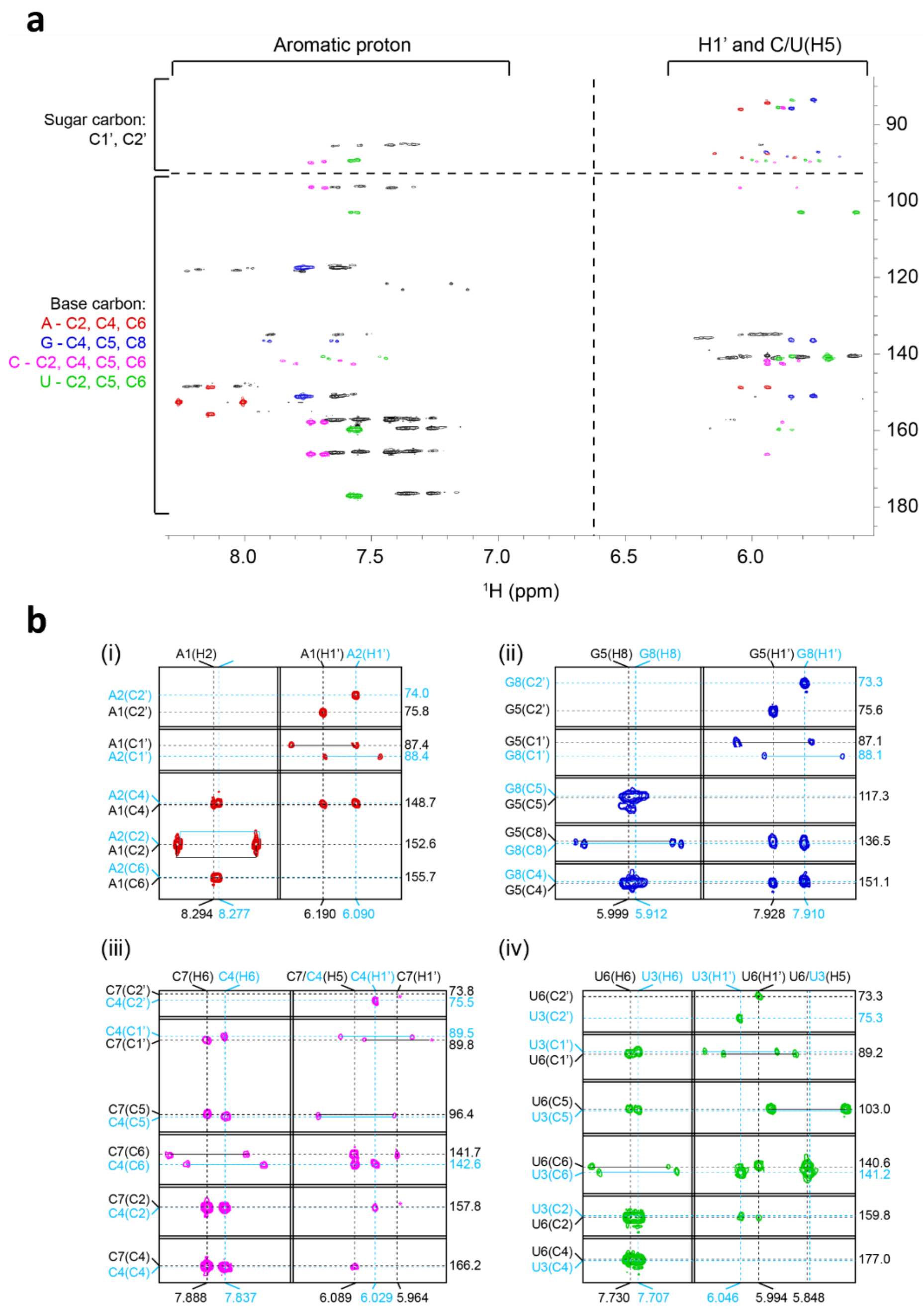

Supplementary Figure 4: (a) ^{1}H-^{13}C Heteronuclear multiple bond correlation (HMBC) NMR spectrum of P. aeruginosa biofilm extracellular nucleic acid (NA) gel isolate at 25°C following alkalinisation (0.1 M NaOD, 10 mg ml^{-1}, 55°C, 2 h). The spectrum is divided into four regions with dashed lines corresponding to the observed interactions between sugar carbons to sugar protons, sugar carbons to base protons, base carbons to sugar protons and base carbons to base protons. Cross-peaks from monoribonucleotides are uniquely colour coded. (b) The detailed assignments based on standard chemical shift values of ribonucleic acids, (i) adenine, (ii) guanine, (iii) cytosine and (iv) uracil. All the assigned cross peaks are marked; the pairs of cross peaks connected by solid lines represents coupled one-bond carbon-proton interactions.

Supplementary Table 1:

Supplementary Table 1: Abundance of individual monoribonucleotides in extracellular nucleic acid (NA) extract from *P. aeruginosa* biofilm as determined by integrating ^{31}P NMR spectral peaks

^{31}P shift (ppm)	Ribonucleotide	Abundance (%)
4.03	3'AMP	18.0 %
4.01	3' GMP	17.2 %
3.90	3' CMP/3' UMP	8.9 %
3.87	3' CMP/3' UMP	11.3 %
3.68	2' CMP/2' UMP	9.5 %
3.66	2' CMP/2' UMP	11.7 %
3.50	2' AMP	10.4 %
3.42	2' GMP	13.0 %

Supplementary Figure 5:

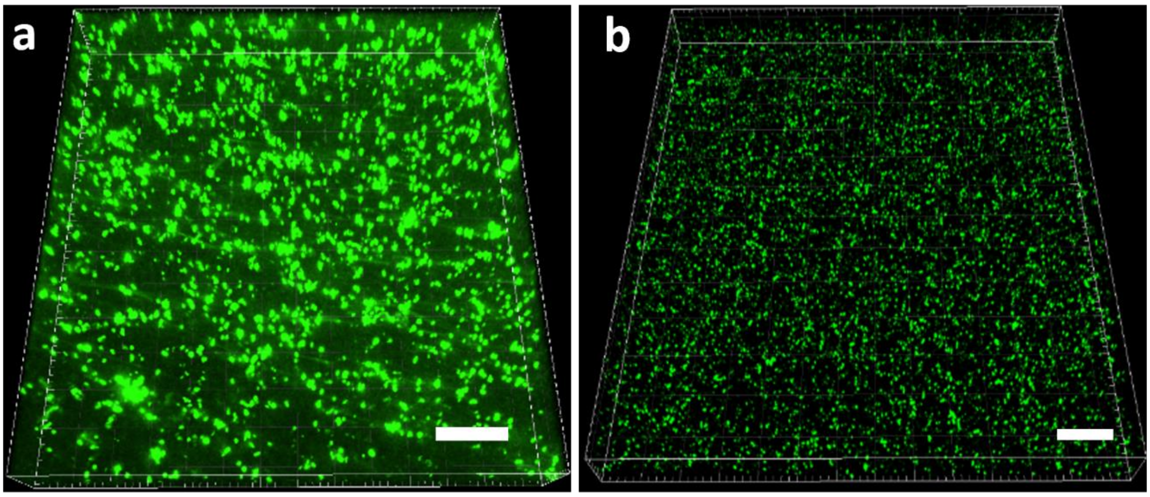

Supplementary Figure 5: Three dimensional (3D) confocal micrographs (n=3) of five-day old *P. aeruginosa* wild type biofilm (a) without any treatment and (b) following alkalinisation, stained with TOTO-1 iodide stain specific for eDNA (Green). The thickness of biofilm is 13 µm. All experiments were performed in biological triplicates. Scale bars represent 10 µm.

Supplementary Figure 6:

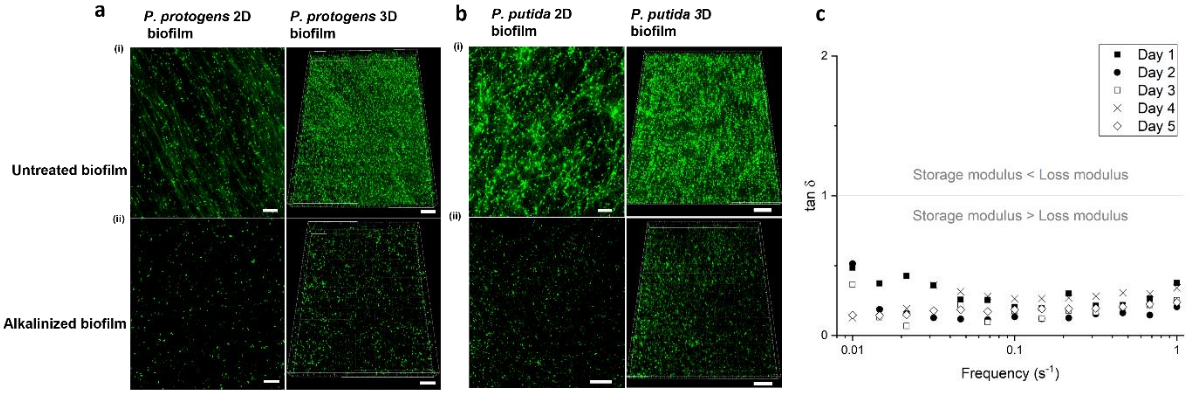

Supplementary Figure 6: Confocal micrographs of five-day old (n=3) (a) *P. protogens* and (b) *P. putida* biofilm without (i) and with alkalinization (ii). The biofilms are stained with TOTO-1 iodide stain specific for eDNA (Green). The thickness of biofilm is 10 µm. All experiments were performed in biological triplicates. Scale bars represent 10 µm. (c) Frequency dependent rheogram (n=3) of different days (Day1- Day 5) of *P. aeruginosa* biofilm growth measuring viscoelasticity. A tan δ > 1 represents fluid behavior, while tan δ < 1 indicates gel behavior. For (c), the biological triplicates are averaged for different days and plotted against frequency. Relevant source data for figure 6c are provided as a source data file.

Supplementary Figure 7:

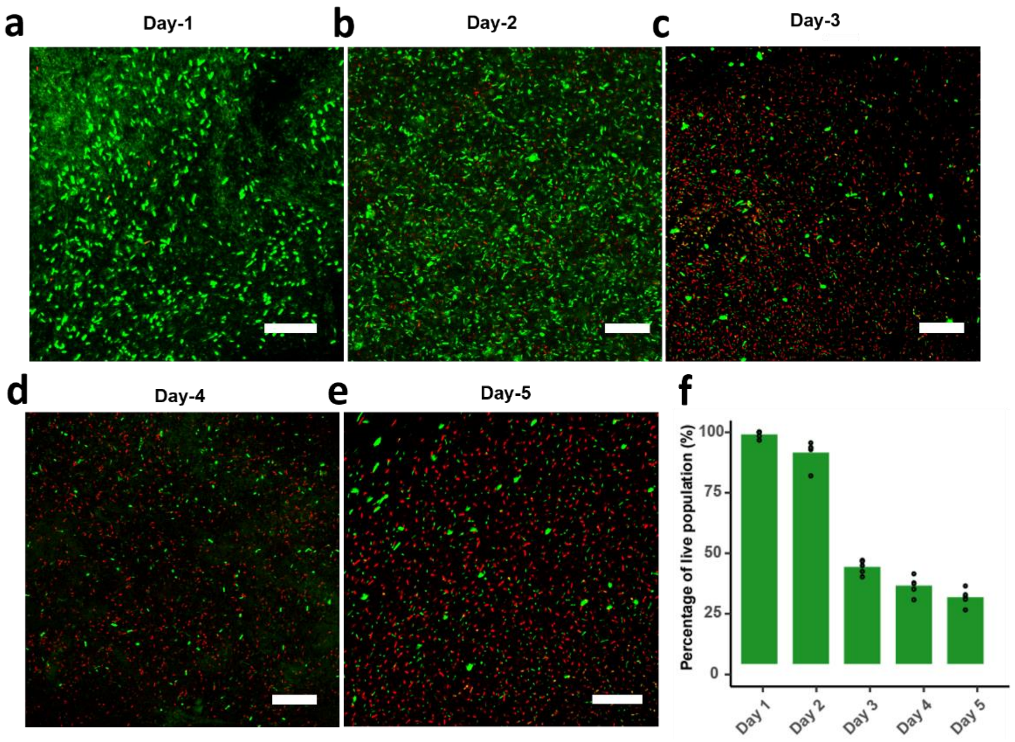

Supplementary Figure 7: Confocal micrographs of live-dead stained (n=5) (Green- Syto9 and Red-Propidium iodide) (a) One-day, (b) two-day old, (c) three-day old, (d) four-day old and (e) five-day old wild-type *P. aeruginosa* PAO1 biofilm. All experiments were performed in biological triplicates. The scale bars represent 10 µm. (f) Fluorescent intensity quantification (n=5) for biofilm viability of one-day to five-day wildtype biofilms as determined using ImageJ fiji software (n=5). Relevant source data for figure 7f are provided as a source data file.

Supplementary Figure 8

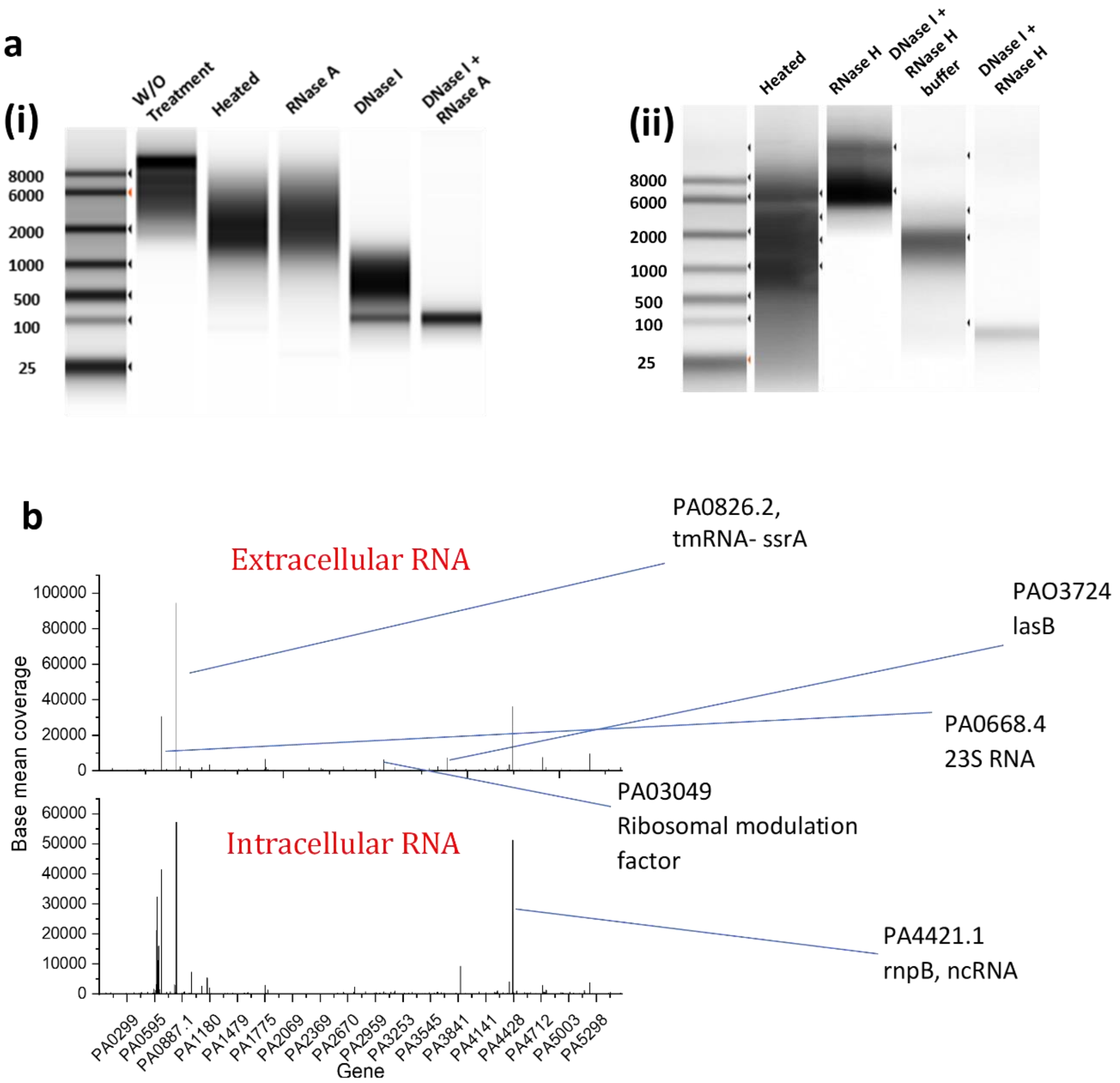

Supplementary Figure 8: (a) (i) Agilent RNA Tape station results of extracellular nucleic acid (NA) purified gel isolate from five-day old *P. aeruginosa* biofilm. Lane 1 is the 1Kbp molecular weight marker, lane 2 is the gel isolate without treatment, lane 3 heating of gel isolate at 55°C, lane 4 is the RNaseA treatment of gel isolate at a concentration of 0.3 mg ml^{-1} for 30 min at 37°C, lane 5 is the DNaseI treatment of the gel isolate at 0.4 mg ml^{-1} at 37°C for 30 min showing loss of high molecular weight DNA, lane 6 is DNaseI followed by RNaseA treatment at 37°C for 1 h showing the complete loss of both lower molecular weight RNA and higher molecular weight DNA. (ii) Agilent RNA Tape station results of extracellular nucleic acid (NA) purified gel isolate from five-day old *P. aeruginosa* biofilm. Lane 1 is the 1Kbp molecular weight marker, lane 2 is the heating of gel isolate at 55°C, lane 3 is the RNaseH treatment of gel isolate at a concentration of 0.3 mg ml^{-1} for 30 min at 37°C, Lane 4 is the

DNaseI followed by RNaseH buffer treatment at 37°C for 30 minutes. Lane 5 is the DNaseI followed by RNaseH treatment at 37°C for 1 h. Molecular weight is indicated in base pairs. (b) The base mean coverage and fold change determined from three biological replicates through RNA sequencing of some mRNAs detected in chromosomal DNA extracted from a 16 h planktonic *P. aeruginosa* culture (i.e. planktonic, as denoted here by Intracellular RNA) and in extracellular nucleic acid (NA) purified gel isolate from five-day old *P. aeruginosa* biofilm (i.e. biofilm matrix, as denoted here by extracellular RNA). The above base mean was calculated by averaging base mean numbers from three biological replicates. Relevant source data for figure 8b are provided as a source data file and deposited to public repository. The link can be found in data availability statement.

Supplementary Table 2a:

Supplementary Table 2a: mRNAs highly detected in extracellular 5-day biofilm matrix compared to chromosomal extract from 16 h overnight culture (i.e. planktonic) from *P. aeruginosa* cells in terms of higher log 2-fold change with a minimum base mean threshold of 30. Relevant source data are provided as a source data file and deposited to public repository. The link can be found in data availability statement.

Locus tag	Base Mean	log2FoldChange	Identity
PA2513	31	7.2	antB
PA3724	2686	5.5	LasB Elastase
PA2381	456	5.2	Hypothetical
PA2250	479	4.3	IpdV
PA3923	426	4.3	Hypothetical
PA2248	485	4.1	bkdA2
PA4726.11	4522	0.6	crcZ
PA0826.2	70253	0.1	Transfer messenger RNA
PA4421.1	44739	-1.2	rnpB
PA0622	20103	-7.5	Probable bacteriophage protein

Supplementary Table 2b:

Supplementary Table 2b: Relative abundance of top 30 mRNAs detected in *P. aeruginosa* biofilm matrix in comparison to planktonic cells with base mean, log 2-fold change and their identity. The log2fold change is the measure of extent of change in gene or transcript's expression between the biofilm matrix sample and planktonic RNA sample. This value is reported on a logarithmic scale to base 2. Relevant source data are provided as a source data file and deposited to public repository. The link can be found in data availability statement.

Locus tag	BaseMean	Log2FoldChang	Identity
PA0826.2	70253	0.02	transfer messenger RNA
PA4421.1	44740	-1.2	rnpB
PA0668.4	37770	-1.2	23S rRNA
PA0622	20103	-7.5	Probable bacteriophage protein
PA0625	15668	-7.4	Hypothetical
PA0620	13348	-7.4	Probable bacteriophage Protein
PA0641	10091	-7.6	Probable bacteriophage protein
PA0636	7032	-7.5	Hypothetical
PA0646	6529	-7.8	Hypothetical
PA0624	6028	-7.8	Hypothetical
PA0623	5964	-7.5	Probable bacteriophage protein
PA5227.1	5945	0.5	6s RNA
PA3866	5933	-7.5	Pyocin S4
PA0621	5193	-7.8	Hypothetical
PA0628	4900	-7.9	Hypothetical
PA0985	4714	-10.1	Pyocin S5
PA4726.11	4521	0.6	crcZ
PA1777	4079	0.29	oprF porin
PA4385	3665	-1.1	GroEL
PA1150	3414	-7.5	Pyocin S2
PA0633	3081	-6.8	Hypothetical
PA0643	3077	-7.1	Hypothetical
PA0639	3014	-7.3	Hypothetical
PA0640	2870	-7.5	Probable bacteriophage protein
PA3724	2686	5.5	Elastase
PA3049	2624	2.3	Ribosomal modulation factor
PA1178	2497	-0.1	oprH
PA1092	2290	-1.3	fliC fimbriae
PA0616	2096	-7.2	Hypothetical

Supplementary Figure 9:

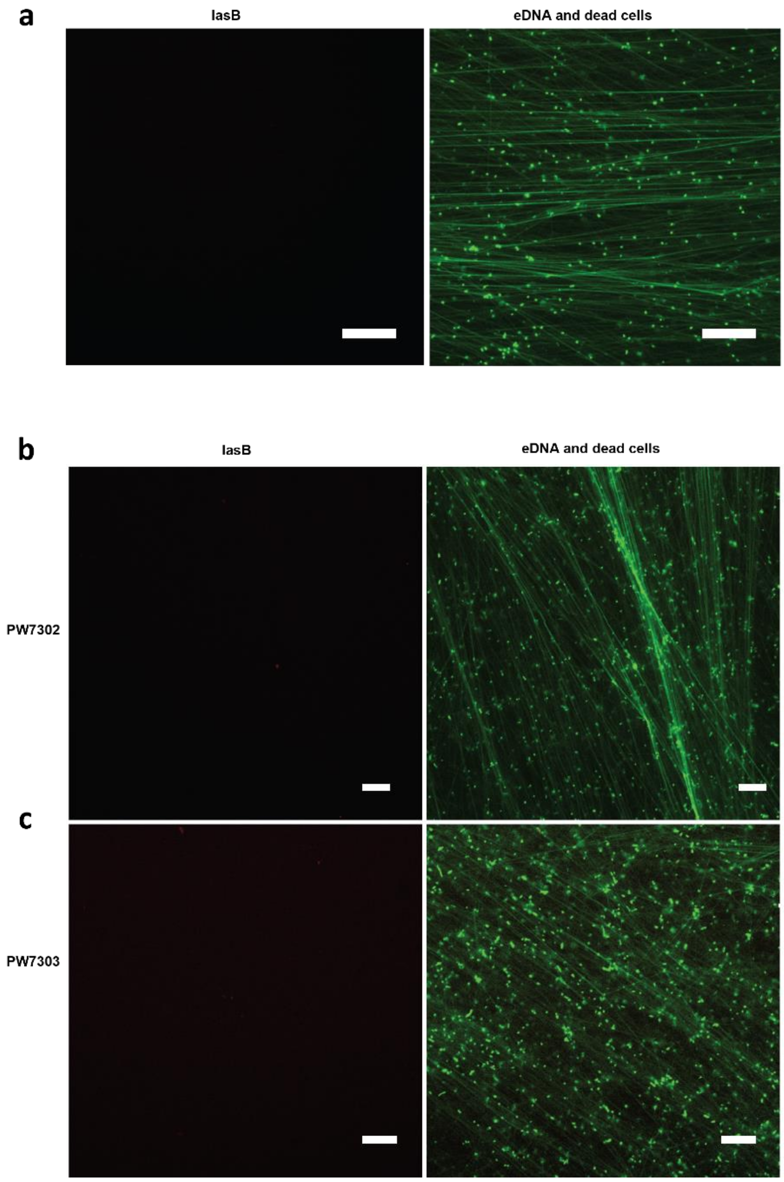

Supplementary Figure 9: smFISH confocal micrographs of five-day old (n=3) *P. aeruginosa* wild type biofilm after 0.1 mg ml^{-1} DNase1 pre-treatment with only (a) FLAP secondary fluorophore (red channel) in the absence of primary probe and TOTO-1 stained eDNA (green channel). This shows the absence of a *lasB* mRNA signal in the eDNA fibres indicating the specificity of secondary FLAP with primary probe. Scale bars represent 10 µm. smFISH confocal micrographs of five-day (n=3) *lasB* transposon mutants of *P. aeruginosa* biofilm after 0.1 mg ml^{-1} DNase1 pre-treatment following staining with *lasB*-specific oligoribonucleotide smFISH probe (Red channel), and eDNA specific TOTO-1 stain (Green channel) showing eDNA fibres without *lasB* mRNA transcripts in the (b) PW7302 (c) and PW7303 (c) mutants respectively. Mutant biofilms were treated with 0.1 mg ml^{-1} DNase I before staining with elastase probe to ensure effective binding to the eDNA fibres. Scale bars represent 10 µm. All experiments were performed in biological triplicates. PW7302 AND PW7303 are transposon mutants derived from mPAO1 isolate of P. aeruginosa strain PAO1. A transposon named ISphoA/hah of 4.83kbp was used to generate these mutant strains with a tetracycline resistant gene [1].

Supplementary Figure 10:

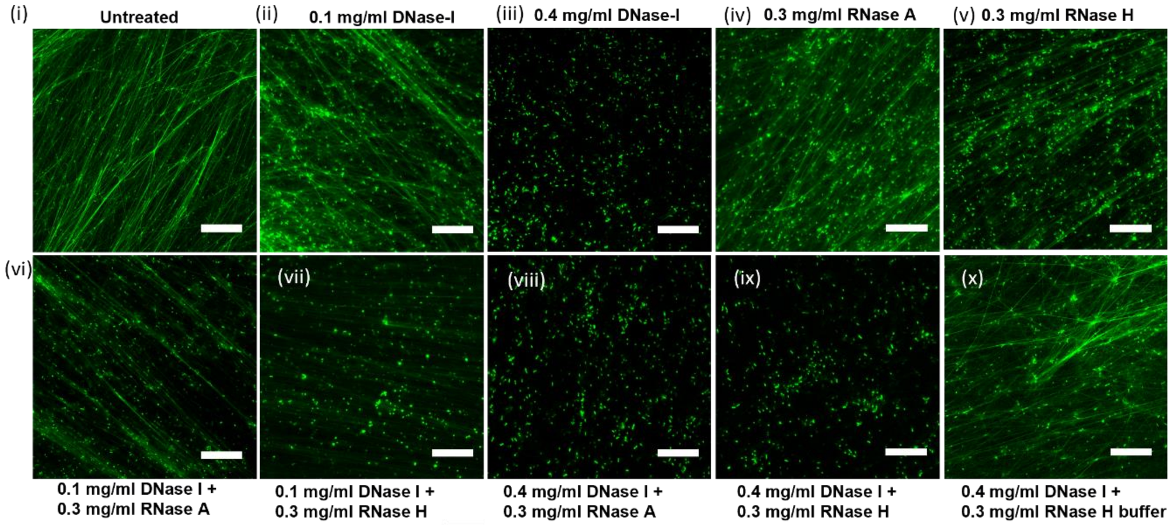

Supplementary Figure 10: Confocal micrograph of five-day old wildtype (n=3) *P. aeruginosa* biofilms with different enzymatic treatments ((i)-(x)) such as 0.1 mg ml^{-1} DNase I for 16 h, 0.4 mg ml^{-1} DNase I, 0.3 mg ml^{-1} RNase A, 0.3 mg ml^{-1} RNase H, double digestions with 0.1 mg ml^{-1} DNase I and 0.3 mg ml^{-1} RNase A, 0.1 mg ml^{-1} DNase I and 0.3 mg ml^{-1} RNase H, 0.4 mg ml^{-1} DNase I and 0.3 mg ml^{-1} RNase A and 0.4 mg ml^{-1} DNase I and 0.3 mg ml^{-1} RNase H and 0.4 mg ml^{-1} DNase I and 0.3 mg ml^{-1} RNase H buffer. All single enzymatic digestions are incubated for 30 minutes, and double digestions are incubated for 1 hour. The digested biofilm is stained with TOTO-1 iodide stain specific for eDNA (Green). All experiments were performed in biological triplicates. Scale bars represent 10 µm.

Supplementary Figure 11:

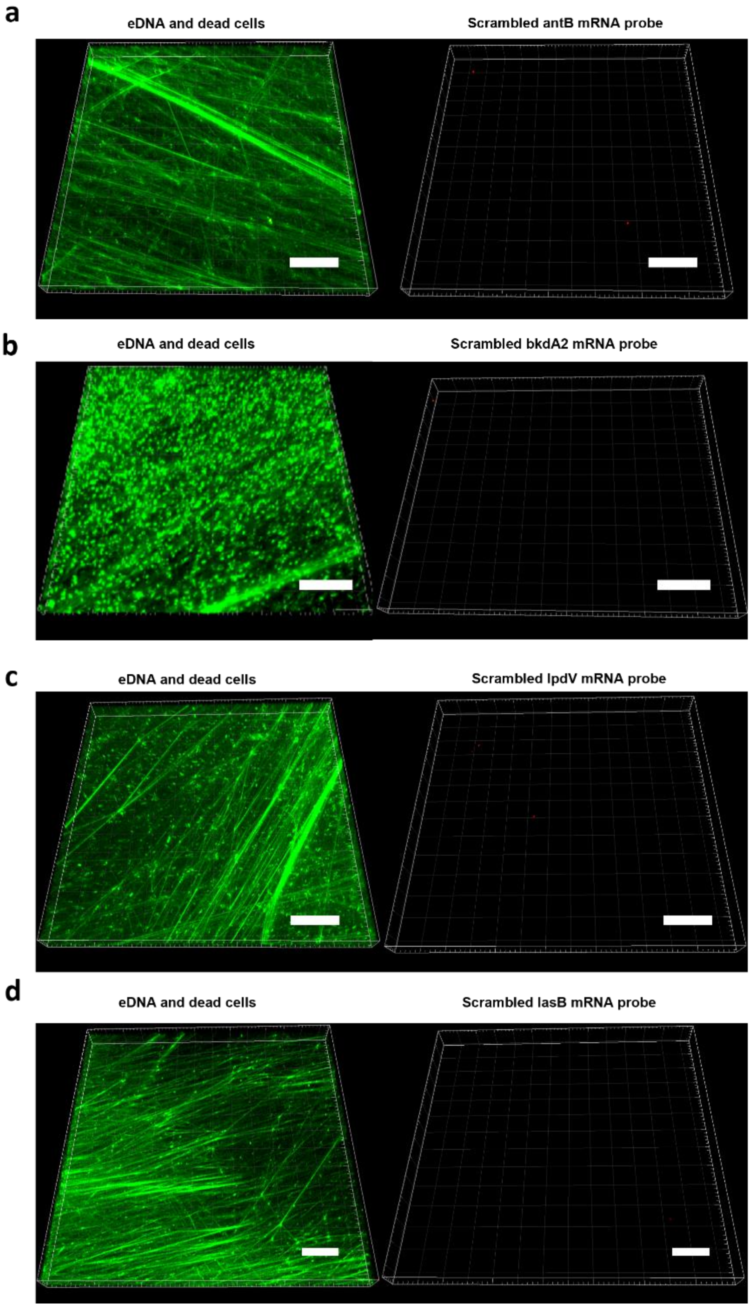

Supplementary Figure 11: Three dimensional (3D) smiFISH confocal micrograph (n=3) of five-day old *P. aeruginosa* biofilms after 0.1 mg ml^{-1} DNase1 pre-treatment following staining with scrambled (a) *antB*, (b) *bkdA-2*, (c) *lpdV* and (d) *lasB* oligoribonucleotide smFISH probes (Red channel). All samples were stained with eDNA specific TOTO-1 stain (Green channel) to show eDNA fibres. All experiments were performed in biological triplicates. The scale bars represent 10 µm.

Supplementary Figure 12:

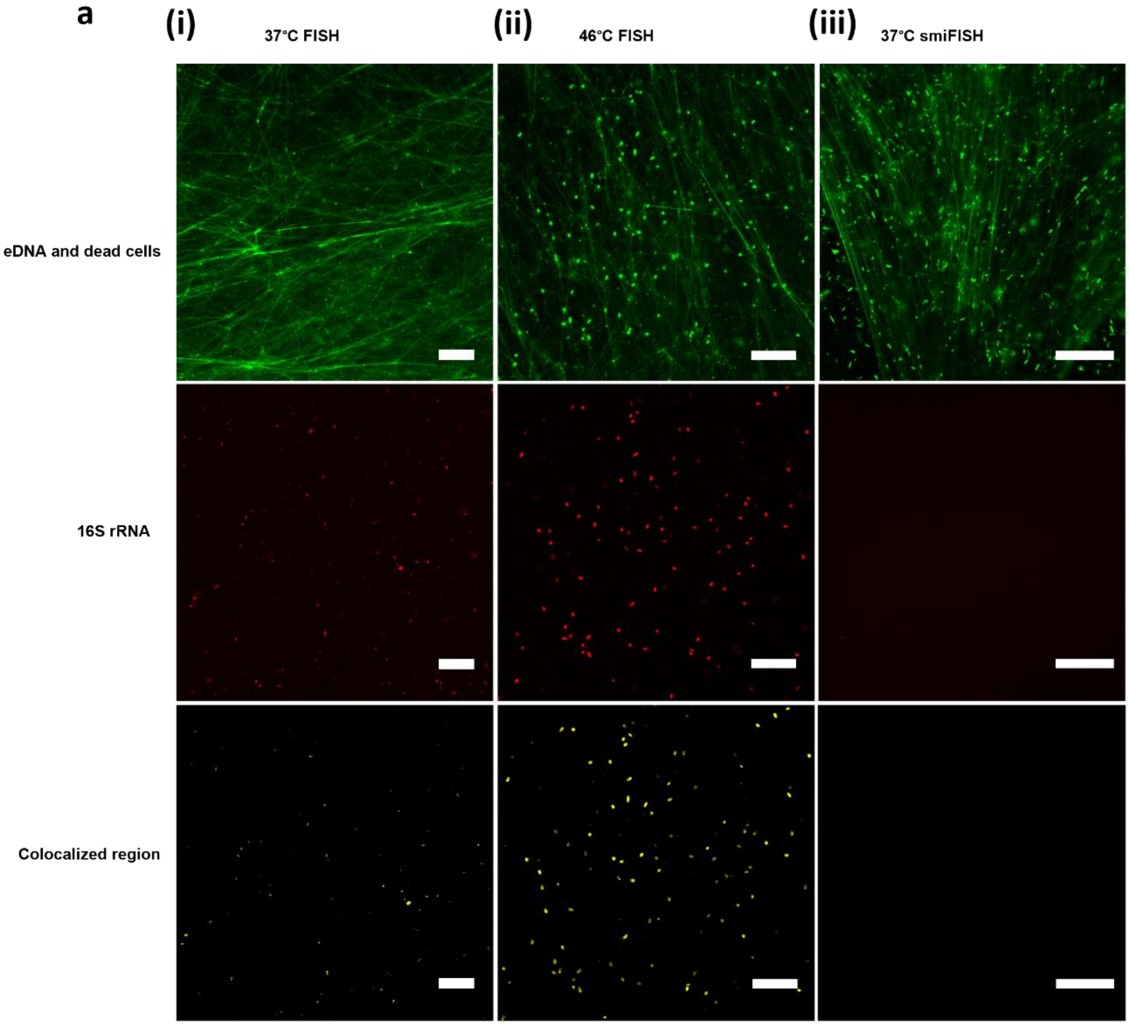

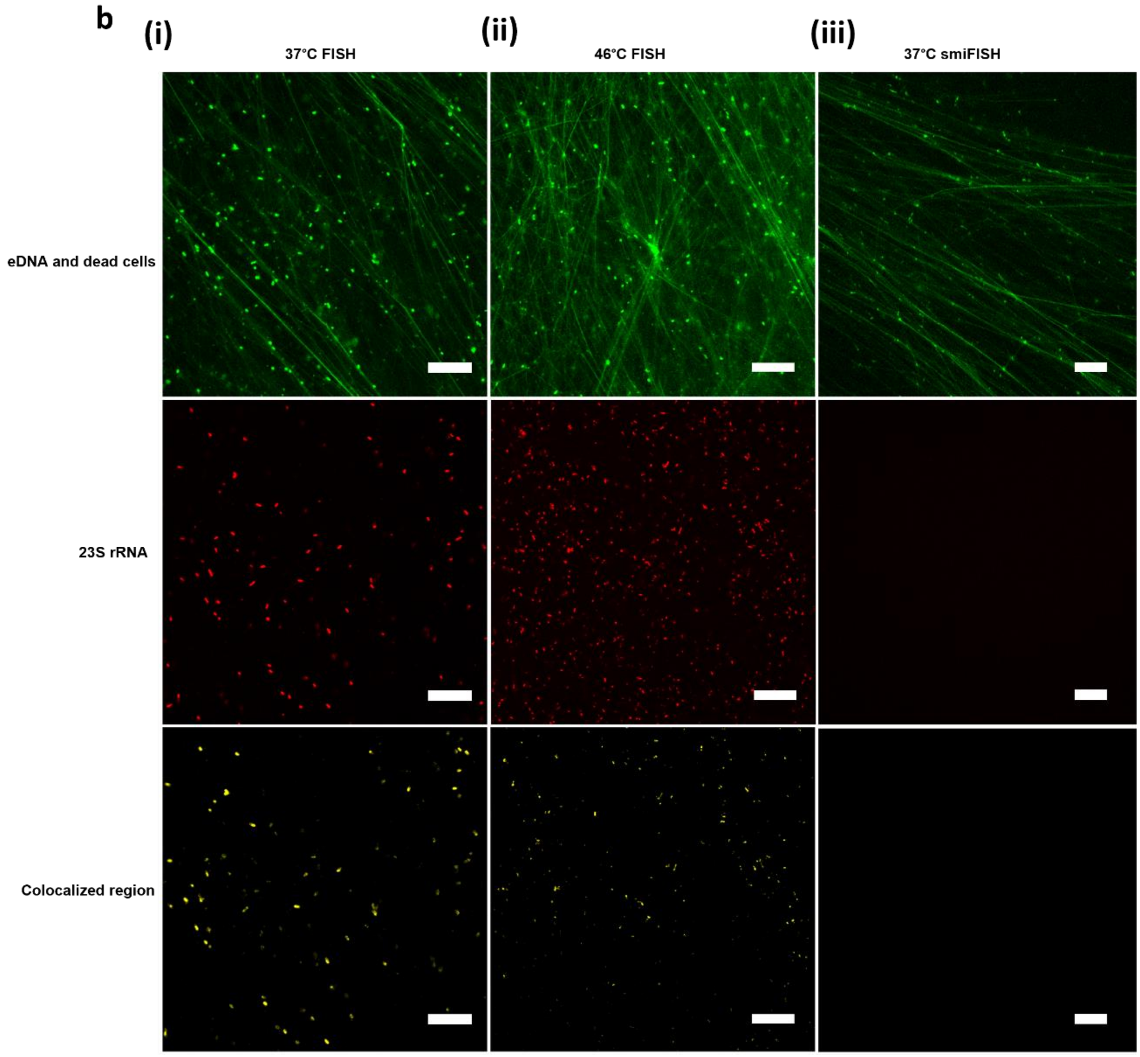

Supplementary Figure 12: Fluorescent in-situ hybridization confocal micrograph (FISH) of 0.1 mg ml^{-1} DNase-I treated five-day old *P. aeruginosa* biofilm (n=3) using (a) 16S rRNA specific probe (red channel) by conventional FISH at (i) 37°C, (ii) 46°C and by (iii) smiFISH at 37°C. Fluorescent in-situ hybridization confocal micrographs of 0.1 mg ml^{-1} DNase-I treated five-day old *P. aeruginosa* biofilm using (b) 23S rRNA specific probe (red channel) at (i) 37°C, (ii) 46°C and (iii) smiFISH at 37°C. eDNA fibres are stained by TOTO-1 specific stain (Green channel). All experiments were performed in biological triplicates. Colocalized channel showing coincidence of ribosomal RNA and eDNA are obtained by imaris x64 software (Yellow channel) software to quantify Mander's coefficient of 0. The scale bars represent 10 µm.

Supplementary Table 3:

Supplementary Table 3: 16s and 23s ribosomal RNA (rRNA) specific probes of Pseudomonas aeruginosa wildtype.

No	Probe Sequence (5'-3')	Name of the probe
1.	GGTAACCGTCCCCCTTGC	PseaerA (16s rRNA PAO1)
2.	TCTCGGCCTTGAAACCCC	PseaerB (23s rRNA PAO1)

Supplementary Figure 13:

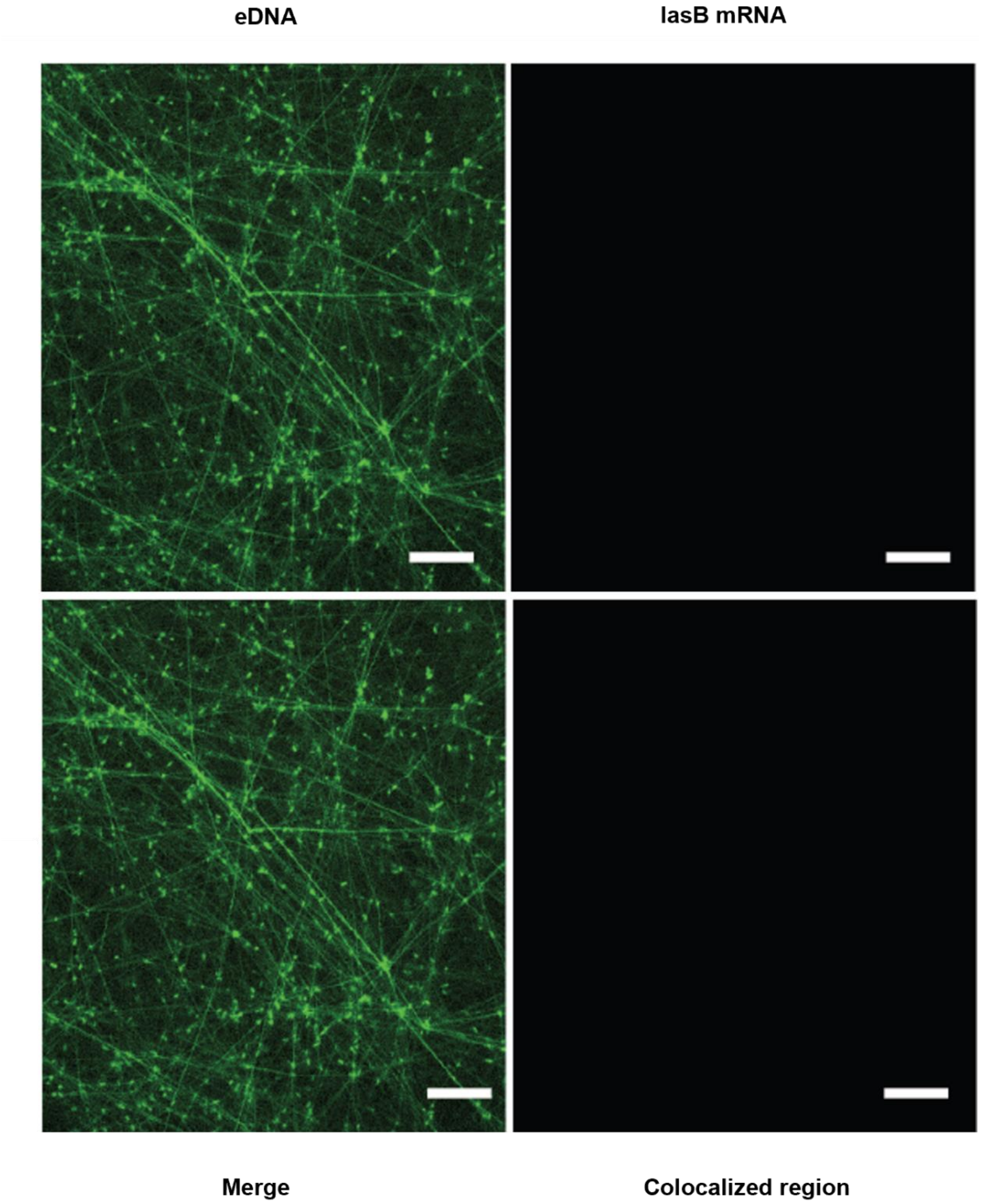

Supplementary Figure 13: smiFISH confocal micrographs (n=3) of one-day old *P. aeruginosa* biofilm after 0.1 mg ml^{-1} DNase I pre-treatment (showing eDNA fibres (Green channel), no *lasB* mRNA signal (Red channel), merge and colocalized region (Yellow channel) calculated using imaris x64 software to quantify Mander's coefficient of 0. All experiments were performed in biological triplicates. Scale bars represent 10 µm.

Supplementary Figure 14:

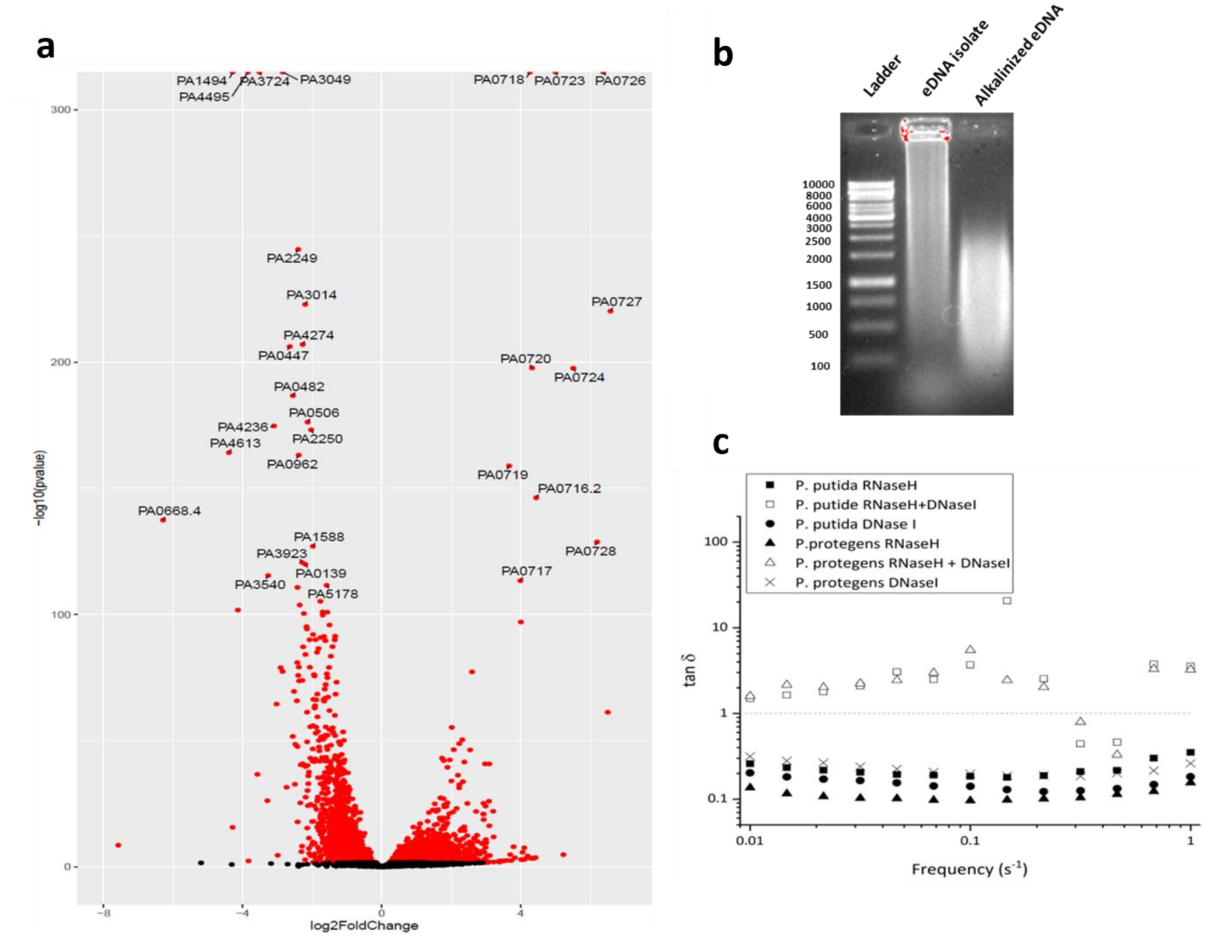

Supplementary Figure 14:

(a) Volcano plot of extracellular RNA sequenced from the biofilm matrix of five-day-old *P. aeruginosa* wildtype biofilm (left) and the *P. aeruginosa* lasB mutant PW7302) (right). mRNA transcripts that are highly abundant in the biofilm matrix of wildtype *P. aeruginosa* (left) are presented on the left and from pw7302 on the right). A higher -log10 p-value indicates a higher probability of the presence of that particular mRNA transcript in the biofilm matrix of either wildtype or lasB mutant of *P. aeruginosa.* Experiments were performed in biological triplicates. (b) Agarose gel electrophoresis (0.8% agarose) of alkalinized extracellular nucleic acid gel isolate (left most lane) and untreated extracellular nucleic acid gel isolate (centre lane) with 1Kbp ladder on the right. Experiments were performed in biological triplicates. (c) Rheogram of five-day old *P. putida and P. protogens* wild-type biofilm (n=3) pretreated with 0.1 mg/ml DNaseI followed by subsequent RNaseH treatment in frequency sweep at 25°C, 0.026mm gap, 0.3 amplitude . A tan δ greater than 1 represents fluid behavior (G' < G''), while tan δ lesser than 1 (i.e. G' > G'') indicates gel behavior, where G' is storage modulus and G'' is loss modulus of elasticity. For (c), the biological triplicates are averaged for both conditions and plotted against frequency. Relevant source data for 14a and c are provided as a source data file.

Supplementary Table 4:

Supplementary table 4: **In silico hybridisation efficiency assay calculator of *lasB* mRNA probe to human elastase and *P. aeruginosa* wildtype *lasB* mRNA**. Negative ΔG value indicate higher efficiency of hybridisation of designed mRNA probe to the target sequence. Positive ΔG value indicate lower possibility of hybridisation reaction between probe and non-target human elastase. Maximum hybridisation efficiency measured was 1 showing complete hybridisation.

Designed *lasB* mRNA Probe Sequence (5'-3')	ΔG overall (Target *P. aeruginosa* elastase) (Kcal/mol)	ΔG overall (Non target Human elastase) (Kcal/mol)	Hybridisation efficiency (Probe and *P. aeruginosa* elastase)	Hybridisation efficiency (Probe and Human elastase)
GATCAGGAAGTCGTTCTTGCCGCGCATA	-22	5	1	1.8*e-13
AGCGGCGAANATAGGCGCCGTTGACCT	-9.1	12.3	0.57	1.1*e-15
CCTGGGCAGCCTTGCNATGGGGAGTTT	-24.5	-7.1	1	0.04
GAGTGGTGCACGTCGATGCCGTTGTAGTA	-29.2	0.4	1	2.62*e-7
CTTCGTTCATTCCGCCTGATTGCCCG	-24.5	13.7	1	1.2*e-16
GCCGAACCAGTCCCGGTACAGTTTGAAC	-25.9	17	1	5.1*e-19
GCTGTTCATGTCGACGGTGATGANACGTTGC	-14.5	9.8	0.99	2.9*e-14
GTACTTGCCGATCTTCTGGTTGCCGC	-26.2	12.4	1	4.7*e-9
CACTGATCGAGCACTTCGCCGGTCTT	-20.6	-1.7	1	3.9*e-6
GTAGACCAGTTGGGCGATGTTGTTCTCGC	-19.9	12.1	1	7.8*e-16
AGGCGGATCACCAGTTCCACTTTGTCA	-22.5	0.2	1	1.83*e-7
TATCCCAGCCCGGCGAATTGGNACCAA	-19.8	8.2	1	4.2*e-13
CACCGCTGCCCTTCTTGATGTCGTAG	-27.4	7.1	1	2.36*e-12

Supplementary Table 5:

Supplementary Table 5: Qubit fluorometry measurement of purified extracellular nucleic gel isolate from *P. aeruginosa* biofilms. The concentration was measured using biological triplicates.

No	DNA concentration (ng/ul)	RNA concentration (ng/ul)
1.	91.2± 5.2	45.6± 3.5

Supplementary Table 6: FLAP hybridization reaction mix (for cycler)

Supplementary Table 6: FLAP hybridization reaction mix (for cycler)

Component	Volume per reaction	Amount
Probe-set*1	2 µL	40 pmol(total DNA)
100 µM FLAP	0.5 µL	50 pmol
10X NEB	1 µL	
H_2O	6.5 µL	

*1 Probe-set: equimolar mixture of primary probes (Refer step *Probe preparation*)

*2 NEB: New England Biolabs Buffer 3 (1X composition: 100mM NaCl, 50mM Tris-HCl, 10mM $MgCl_2$, pH 7.9

Supplementary table 7:

Supplementary table 7: Incubation conditions of *FLAP* hybridization reaction mix in a PCR thermocycler.

1cycle	85°C	3 min
1cycle	65°C	3 min
1cycle	25°C	3 min

Supplementary Table 8:

Supplementary Table 8: *In situ* hybridization reaction *Mix 1* and *Mix 2* (for two coverslips 22 x 22 mm)

Mix 1	Volume
20x SSC	5µL
20 µg/µl E. coli tRNA	1.7 µL
100 % Formamide	15 µl
Flap- structured duplexes	2 µl
Water	26.2 µl
**Mix 2**	**Volume**
20 mg/ml RNAse-free BSA	1 µL
200 mM VRC	1 µL
40 % dextran sulfate	26.5 µl
Water	21.5 µl

Supplementary References

1. Jacobs MA, *et al.* Comprehensive transposon mutant library of Pseudomonas aeruginosa. *Proceedings of the National Academy of Sciences* **100**, 14339-14344 (2003).
