# Supplementary material for: RNA is a key component of extracellular DNA networks in Pseudomonas aeruginosa biofilms
Source: Nat Commun. 2023 Nov 27;14:7772. doi: 10.1038/s41467-023-43533-3 (PMC10682433; doi:10.1038/s41467-023-43533-3)
Supplement: Supplementary file 3 — Description of Additional Supplementary Files [file 41467_2023_43533_MOESM3_ESM.pdf]

Description of Additional Supplementary Files:

Supplementary Data 1: Primary and secondary oligoribonucleotide sequences used to observe specific mRNA transcripts in Pseudomonas aeruginosa PAO1 static biofilm and clinical sputum samples by smiFISH confocal microscopy.
