# Supplementary material for: RNA is a key component of extracellular DNA networks in Pseudomonas aeruginosa biofilms
Source: Nat Commun. 2023 Nov 27;14:7772. doi: 10.1038/s41467-023-43533-3 (PMC10682433; doi:10.1038/s41467-023-43533-3)
Supplement: Supplementary file 5 — Reporting Summary [file 41467_2023_43533_MOESM5_ESM.pdf]

nature portfolio

Corresponding author(s): Thomas William Seviour

Last updated by author(s): Nov 8, 2023

Reporting Summary

Nature Portfolio wishes to improve the reproducibility of the work that we publish. This form provides structure for consistency and transparency in reporting. For further information on Nature Portfolio policies, see our Editorial Policies and the Editorial Policy Checklist.

Statistics

For all statistical analyses, confirm that the following items are present in the figure legend, table legend, main text, or Methods section.

n/a	Confirmed	
☐	☒	The exact sample size (n) for each experimental group/condition, given as a discrete number and unit of measurement
☐	☒	A statement on whether measurements were taken from distinct samples or whether the same sample was measured repeatedly
☐	☒	The statistical test(s) used AND whether they are one- or two-sided<br>*Only common tests should be described solely by name; describe more complex techniques in the Methods section.*
☒	☐	A description of all covariates tested
☒	☐	A description of any assumptions or corrections, such as tests of normality and adjustment for multiple comparisons
☐	☒	A full description of the statistical parameters including central tendency (e.g. means) or other basic estimates (e.g. regression coefficient) AND variation (e.g. standard deviation) or associated estimates of uncertainty (e.g. confidence intervals)
☐	☒	For null hypothesis testing, the test statistic (e.g. F, t, r) with confidence intervals, effect sizes, degrees of freedom and P value noted<br>*Give P values as exact values whenever suitable.*
☒	☐	For Bayesian analysis, information on the choice of priors and Markov chain Monte Carlo settings
☒	☐	For hierarchical and complex designs, identification of the appropriate level for tests and full reporting of outcomes
☐	☒	Estimates of effect sizes (e.g. Cohen's d, Pearson's r), indicating how they were calculated

Our web collection on statistics for biologists contains articles on many of the points above.

Software and code

Policy information about availability of computer code

Data collection

No Software used in this study for data collection.

Data analysis

System:
Windows 10 Education, version 21H2,
OS BUILD: 19044.1826
RNA sequencing Packages:
1) Trim galore version 0.6.7- adapter trimming of RNA
2) SortMeRNA version 4.3.4- Remove ribosomal RNA
3) SILVA 16S rRNA v138 database- Remove ribosomal RNA
4) Bowtie2 version version 2.4.4- Mapping to reference genome
5) SAMtools version version 1.4 - SAM file conversion of mapped reads
6) HTSeq count version 1.99.2 - Counting/quantifying mapped reads
7) Deseq2 version 3.14 - Differential expression of genes between samples
FISH probe design:
1) R version 4.2.1 (Funny-Looking Kid)
2) Oligostan.r (Additional packages required- seqinr, Zoo)

For manuscripts utilizing custom algorithms or software that are central to the research but not yet described in published literature, software must be made available to editors and reviewers. We strongly encourage code deposition in a community repository (e.g. GitHub). See the Nature Portfolio guidelines for submitting code & software for further information.

Data

Policy information about availability of data

All manuscripts must include a data availability statement. This statement should provide the following information, where applicable:

- Accession codes, unique identifiers, or web links for publicly available datasets
- A description of any restrictions on data availability
- For clinical datasets or third party data, please ensure that the statement adheres to our policy

The source data file supporting the findings of this study are available within the paper and its Supplementary Information. The RNA sequencing data analysis from PAO1 and metagenomic data of clinical sputum sample has been deposited to sequence read archive (SRA) with a BioProject accession number PRJNA890467 and PRJNA595703 respectively. The data can be found from: https://www.ncbi.nlm.nih.gov/sra.

Research involving human participants, their data, or biological material

Policy information about studies with human participants or human data. See also policy information about sex, gender (identity/presentation), and sexual orientation and race, ethnicity and racism.

Reporting on sex and gender	The information asked is not relevant to our study
Reporting on race, ethnicity, or other socially relevant groupings	The information asked is not relevant to our study
Population characteristics	Three clinical airway specimens (as spontaneously expectorated sputum) were obtained from three patients with severe post-infection bronchiectasis (i.e. Bronchiectasis Severity Index score 16) during a period of clinical stability. Bronchiectasis was confirmed by high resolution computed tomography (HRCT) and clinical stability defined as the absence of new symptoms and/or change in bronchiectasis therapy in the preceding four-week period prior to sampling.<br>Nondiseased (healthy) individual had no active or past history of any respiratory or other medical disease and normal spirometry measured in accordance with European Respiratory Society/American Thoracic Society criteria. Nondiseased individual was free from any exposure to inhaled medications or antibiotic use in the preceding 12-month period.
Recruitment	Recruitment was carried out during outpatient attendance at Singapore General Hospital, Singapore. Patients were clinically stable, which was defined as the absence of new symptoms and no change to bronchiectasis therapy in the preceding 4 weeks and complied with the inclusion criteria of confirmed radiological bronchiectasis on HRCT. Patients were excluded if they had a concurrent chronic respiratory disease (asthma or COPD) as their primary diagnosis, had active mycobacterial disease or an active infection (necessitating acute use of antibiotics). Patients who were pregnant or breastfeeding, on chemotherapy or on systemic corticosteroids in 4 weeks preceding recruitment were also excluded. As standard recruitment practices were used, no bias was introduced.<br>Nondiseased (healthy) individual was recruited through an established voluntary exercise program at Nanyang Technological University, Singapore.
Ethics oversight	This study was approved by the Institutional Review Boards (IRBs) of the participating hospital and institution and written informed consent was obtained from the participant. Reference numbers pertaining to ethical approvals at each site was as follows: CIRB 2017/2109 (Singapore General Hospital), IRB-2017-05-035 (Nanyang Technological University, Singapore).<br>Reference number pertaining to ethical approval for non-diseased healthy patient sample collection was as follows: NTU IRB-2017-07-023

Note that full information on the approval of the study protocol must also be provided in the manuscript.

Field-specific reporting

Please select the one below that is the best fit for your research. If you are not sure, read the appropriate sections before making your selection.

[x] Life sciences [ ] Behavioural & social sciences [ ] Ecological, evolutionary & environmental sciences

For a reference copy of the document with all sections, see nature.com/documents/nr-reporting-summary-flat.pdf

Life sciences study design

All studies must disclose on these points even when the disclosure is negative.

Sample size	This was a detailed study on the EPS of Pseudomonads. The focus was on Pseudomonas aeruginosa, although we also looked at Pseudomonas protogens and Pseudomonas putida (i.e. 3 Pseudomonads). The biofilm model system was a 500 mL system. The volume was determined by how much was needed for us to collect 10 mg NA gel isolate. Even though this volume is in excess of that required to meet the minimum amount for sequencing (400 ng) the volume was kept constant between NA extraction assays for NMR, and for sequencing.

Data exclusions	No data were excluded from the study.
Replication	NA gel was isolated for NMR analysis from three different biofilm growth models, on three different time points, approximately evenly spaced over 12 months. For sequencing, RNA was extracted from three biological replicates cultivated at the same time. For microscopy, biofilms samples in triplicates across different experimental parameters were imaged once every week. Rheology measurements were performed in biological triplicates across different experimental parameters once in two weeks.
Randomization	A single strain of Pseudomonas aeruginosa (i.e. PAO1) was selected as the focus of the study because it is a common laboratory biofilm reference strain (https://www.frontiersin.org/articles/10.3389/fmicb.2022.1023523/full= https://www.frontiersin.org/articles/10.3389/fmicb.2022.1023523/full). Sputum samples were selected on the basis of high P. aeruginosa load (i.e. >95).
Blinding	No blinding was required because the possibility of biases affecting the interpretation of results (e.g. with imaging) was managed by means of controls and statistical analysis

Reporting for specific materials, systems and methods

We require information from authors about some types of materials, experimental systems and methods used in many studies. Here, indicate whether each material, system or method listed is relevant to your study. If you are not sure if a list item applies to your research, read the appropriate section before selecting a response.

Materials & experimental systems

n/a	Involved in the study
☒	☐ Antibodies
☒	☐ Eukaryotic cell lines
☒	☐ Palaeontology and archaeology
☒	☐ Animals and other organisms
☒	☐ Clinical data
☒	☐ Dual use research of concern
☒	☐ Plants

Methods

n/a	Involved in the study
☒	☐ ChIP-seq
☒	☐ Flow cytometry
☒	☐ MRI-based neuroimaging

Plants

Seed stocks	*Report on the source of all seed stocks or other plant material used. If applicable, state the seed stock centre and catalogue number. If plant specimens were collected from the field, describe the collection location, date and sampling procedures.*
Novel plant genotypes	*Describe the methods by which all novel plant genotypes were produced. This includes those generated by transgenic approaches, gene editing, chemical/radiation-based mutagenesis and hybridization. For transgenic lines, describe the transformation method, the number of independent lines analyzed and the generation upon which experiments were performed. For gene-edited lines, describe the editor used, the endogenous sequence targeted for editing, the targeting guide RNA sequence (if applicable) and how the editor was applied.*
Authentication	*Describe any authentication procedures for each seed stock used or novel genotype generated. Describe any experiments used to assess the effect of a mutation and, where applicable, how potential secondary effects (e.g. second site T-DNA insertions, mosiacism, off-target gene editing) were examined.*
